# Third-generation Sequencing Reveals Extensive Polycistronism and Transcriptional Overlapping in a Baculovirus

**DOI:** 10.1038/s41598-018-26955-8

**Published:** 2018-06-05

**Authors:** Norbert Moldován, Dóra Tombácz, Attila Szűcs, Zsolt Csabai, Zsolt Balázs, Emese Kis, Judit Molnár, Zsolt Boldogkői

**Affiliations:** 1 Department of Medical Biology, Faculty of Medicine, University of Szeged, Szeged, 6720 Hungary; 2 Solvo Biotechnology, Szeged, 6720 Hungary

## Abstract

The *Autographa californica multiple nucleopolyhedrovirus* (AcMNPV) is an insect-pathogen baculovirus. In this study, we applied the Oxford Nanopore Technologies platform for the analysis of the polyadenylated fraction of the viral transcriptome using both cDNA and direct RNA sequencing methods. We identified and annotated altogether 132 novel transcripts and transcript isoforms, including 4 coding and 4 non-coding RNA molecules, 47 length variants, 5 splice isoforms, as well as 23 polycistronic and 49 complex transcripts. All of the identified novel protein-coding genes were 5′-truncated forms of longer host genes. In this work, we demonstrated that in the case of transcript start site isoforms, the promoters and the initiator sequence of the longer and shorter variants belong to the same kinetic class. Long-read sequencing also revealed a complex meshwork of transcriptional overlaps, the function of which needs to be clarified. Additionally, we developed bioinformatics methods to improve the transcript annotation and to eliminate the non-specific transcription reads generated by template switching and false priming.

## Introduction

The *Autographa californica multiple nucleopolyhedrovirus* (AcMNPV) is an insect virus belonging to the *Alphabaculovirus* genus of *Baculoviridae* family^1^. The lifecycle of AcMNPV includes two distinct forms: the budded virus (BV) is released from the infected cells first, while the occlusion-derived virus (ODV) is released later. ODV is responsible for the primary infection, while the BV infects the host cells during the secondary infection^2^.

The AcMNPV is a double-stranded DNA virus with a 133 kilobase-pair (kbp)-long circular genome encompassing 156 closely-spaced open reading frames (ORFs). The viral genome is complex with respect to the transcription^3^. The baculovirus genes are expressed in three distinct phases: early (E), late (L) and very late (VL); the early genes can be subdivided into immediate early (IE) and delayed early (DE) genes^2^. The promoters of IE and E genes commonly harbour a canonical TATA motif that are recognized by the host RNA polymerase II, and their transcription starts at an early initiator CAGT sequence^4^. On the other hand, the L and VL transcripts tend to bind to a late initiator sequence (LIS) harbouring a TAAG motif recognized by viral RNA polymerase (RNAP), which starts the transcription from the second nucleotide of the motif ^3,5^. The E transcripts contain the consensus AAUAAA or AUUAAA polyadenylation (PA) signal (PAS), and their PA tail formation is carried out by the nuclear polyadenylation machinery of the host cell. On the other hand, it is assumed that the L and VL transcripts do not require the consensus PAS. Using an *in vitro* system, Jin and Guarino^6^ demonstrated that the viral RNAP enzyme lacking the carboxy-terminal domain, an essential part in the recruitment of the polyadenylation apparatus, can perform a non-templated addition of adenosines after terminating at a T-rich sequences. Baculoviruses are commonly used as gene delivery vectors in insect cell systems^7^ for the expression of recombinant proteins^8^ or biopesticides^9^.

Next-generation sequencing (NGS) techniques have proved to be useful for discovering novel genes and characterizing their expressions^10–12^. While these platforms are highly accurate and produce a massive amount of output data, they are inefficient for identifying polycistronic and complex transcripts, and for distinguishing between RNA length and splice isoforms^13^ and between overlapping transcripts.

Third generation sequencing (TGS) techniques can surmount these shortcomings by their ability to determine the full-length sequence of the RNA molecules^14–16^ using cDNA sequencing (cDNA-Seq) or direct (d)RNA sequencing (dRNA-Seq). The Oxford Nanopore Technologies (ONT) MinION platform is based on the passage of unlabelled DNA or RNA molecules through a protein channel present on a synthetic membrane, which is regulated by a motor protein^17^. The passage of the nucleotides causes changes in the ionic flow through the nanopore, which can be associated with the specific nucleotide^18^. Theoretically, the ONT technology has no upper limit regarding the read length, but currently falls short of its competitors, especially compared to the Pacific Biosciences (PacBio) platform, in terms of the precision of base identification^19^. However, in the case of well-annotated genomes and high data coverage, this technology is optimal for global transcriptome analysis. Both NGS and TGS technologies use reverse transcription (RT) and PCR for library preparation, which can produce false products (false splice sites as well as false 5′ and 3′ ends) through template switching^20,21^, or through false priming^22^. The dRNA-Seq represents a useful approach for the elimination of these artefacts. However, besides the relatively high false INDEL and base substitution ratio, dRNA sequencing is afflicted by two other innate flaws, which should be taken into account when searching for novel transcript variants. In our previous study^16^, we observed that the 5′ ends of direct RNA sequencing reads were on average 23 bp shorter than the actual transcription start sites (TSSs). We assume that this could be the result of the premature release of the RNA strand by the motor protein, causing a rapid transition of the RNA molecule through the nanopore, and thus cause a perturbation of the base calling near the 5′ end. Our other observation was that the PA tails were miscalled as CT-rich regions^16^ for which the reason may be that the dRNA-Seq base caller algorithm is perturbed by the presence of a DNA adaptor ligated to the PA sequences.

Our research group investigated various viruses using PacBio sequencing alone^23–25^ and using a multiplatform (PacBio, ONT and Illumina) sequencing approach^16^. The advantage of the PacBio platform over ONT sequencing was the very high accuracy, while the ONT was found to be superior in identifying transcripts with sizes ranging from 200 to 800 bp.

Previous studies of the AcMNPV transcriptome using microarray^26^ and real-time RT-PCR^27^ analysis focused mainly on the expression dynamics, while a study using 5′ and 3′ rapid amplification of cDNA ends (RACE) and Illumina sequencing of 5′ capped RNA described many of the potential TSSs and transcription end sites (TESs)^3^. Despite the high precision of these methods, they are unable to uncover the transcriptome complexity in its entirety, they fall short especially in the identification of transcript isoforms, multigenic RNA molecules and transcript overlaps.

In this study, we used the ONT MinION long-read real-time cDNA sequencing for confirming the 5′ and 3′ ends of already known AcMNPV transcripts with base-pair precision, and for identifying novel RNA molecules, including putative protein-coding and non-coding transcripts, TSS and TES isoforms, as well as polycistronic and complex transcripts. Additionally, we also applied a dRNA-Seq technique in order to exclude the artefacts produced by PCR and RT, and for confirming the existence of the RNA molecules identified by cDNA-Seq.

## Materials and Methods

### Cells and viral infection

The baculovirus AcMNPV, used in this study expresses the *lacZ* gene, which was inserted in the promoter region of the *polh* gene (βgal-AcMNPV). This virus was propagated on the Sf9 cell line (kindly provided by Ernő Duda Jr., Solvo Biotechnology, Hungary). Cells were cultivated in 200 ml of GIBCO Sf-900 II SFM insect cell medium (Thermo Fisher Scientific) in a Corning spinner flask (Merck) set to 70 rpm at 26 °C, and were infected with a viral titre of 2 multiplicity of infection (MOI = plaque-forming units per cell). A five ml sample was measured and centrifuged at 2,000 rpm at 4 °C at nine consecutive time points (0 h, 1 h, 2 h, 4 h, 6 h, 16 h, 24 h, 48 h and 72 h), followed by washing with PBS and centrifuged again. Pellets were stored at −80 °C until use. The samples were mixed for the sequencing analysis.

### RNA purification

Total RNA was isolated using the Nucleospin RNA Kit (Macherey-Nagel) according to the manufacturer’s guidance. In short, infected cells were collected by centrifugation and the cell membrane was disrupted by the addition of lysis buffer (derived from the kit). Genomic DNA was digested by treatment with RNase-free rDNase solution (supplied with the kit). Samples were eluted in a total volume of 50 μl nuclease free water. To eliminate residual DNA contamination, samples were treated with TURBO DNA-free Kit (Thermo Fisher Scientific). The RNA concentration was measured using a Qubit 2.0 Fluorometer, using the Qubit RNA BR Assay Kit (Thermo Fisher Scientific). The poly(A)+ RNA fraction was isolated from the samples using the Oligotex mRNA Mini Kit (Qiagen). An mRNA mix was prepared by using 10 μl from each time point. RNA samples were stored at −80 °C until use.

### Oxford Nanopore MinION sequencing

*The* ‘*strand switching cDNA by ligation*’ *approach* The cDNA library was prepared using the Ligation Sequencing kit (SQK-LSK108; Oxford Nanopore Technologies) following the 1D strand switching cDNA by ligation protocol. Briefly: single-stranded (ss)cDNA synthesis was carried out using 50 ng poly(A)+ RNA, SuperScript IV Reverse Transcriptase (Thermo Fisher Scientific) and anchored adapter-primer with (VN)T_20_ nucleotides (nts; supplied by the kit). A 5′ adapter sequence with three O-methyl-guanine RNA bases was added for the facilitation of strand switching. PCR was carried out using Kapa HiFi DNA polymerase (Kapa Biosystems) and the primers supplied in the kit. End repair was conducted using NEBNext End repair/dA-tailing Module (New England Biolabs) followed by adapter ligation using adapters (supplied in the kit) and NEB Blunt/TA Ligase Master Mix (New England Biolabs). The cDNA sample was purified between each step using Agencourt AMPure XP magnetic beads (Beckman Coulter) and the library concentration was determined using a Qubit 2.0 Fluorometer (through use of the Qubit (ds)DNA HS Assay Kit (Thermo Fisher Scientific). Samples were loaded on R9.4 SpotON Flow Cells, and base calling was performed using Albacore v1.2.6.

*The direct RNA sequencing approach* Libraries were prepared using the Direct RNA Sequencing Kit (SQK-RNA001; Oxford Nanopore Technologies). The first strand cDNA was synthesized by SuperScript IV Reverse Transcriptase (Thermo Fisher Scientific) using an RT adapter with T_10_ nts and the mRNA mix. Adapters (supplied by the kit) were ligated using T4 DNA ligase (New England Biolabs). The RNA-DNA hybrid was purified between each step by using Agencourt AMPure XP magnetic beads (Beckman Coulter), and then treated with RNaseOUT Recombinant Ribonuclease Inhibitor (Thermo Fisher Scientific). Sample concentration was determined using a Qubit 2.0 Fluorometer and the Qubit DNA HS Assay Kit (Thermo Fisher Scientific). Libraries were loaded on R9.4 SpotON Flow Cells. Albacore software (v1.2.6) was used for base calling.

### Transcript annotation, visualisation and *in silico* analysis

Reads of both sequencing approaches were aligned to the circularized genome of AcMNPV strain E2 (GeneBank accession: KM667940.1) and the host cell genome (*Spodoptera frugiperda* isolate Sf9; BioProject accession: PRJNA380964) using GMAP v2017-04-24^28^. For the annotation of TSSs, AcMNPV alignments were analysed using the Smith-Waterman algorithm, with a match cost of +2, a mismatch cost of −3, a gap open cost of −3, and a gap extension cost of −2. The last 16 nucleotides of the MinION 5′ adapter were aligned to a window of −10 nucleotides (nt) upstream and +30 nt downstream of the first mapped nucleotide of the read. Reads with a score of less than 17 were considered as putative false 5′ ends caused by strand switching or non-specific priming. A position was considered the 5′ end of a read if the last four nucleotides of the MinION 5′ adapter were detected in a window of −2 upstream to +3 downstream of the first mapped nucleotide. The use of this 5-nucleotide window is necessary due to the varying numbers of base called G letters, caused by the sequencer’s homopolymer error, and the homology uncertainty of the mapper. A 5′ end position was considered TSS if the number of reads starting at this position was significantly higher than at other nucleotides in the region surrounding this start position. For this the Poisson-probability (Poisson [k_0_; λ]) of k_0_ read starting at a given nucleotide in the −50 nt to +50 nt window from each local maximum was calculated with $\lambda =\frac{\sum _{i=-50}^{50}{k}_{i}}{101}$.

The 5′ ends of the longest low-abundance reads were individually inspected using Integrative Genomics Viewer (IGV)^29^. Poly(A) tails were defined as soft-clipped homopolymer A or homopolymer T stretches of at least 15 nts, or in some cases for the ONT direct RNA sequencing data soft-clipped CT-rich or GA-rich regions. The latter is required because of the base caller error of the dRNA-Seq^16^. The last mapped nt upstream of a poly(A) tail was considered as TES if at least 10 mapped reads ended in the given position, except when the TES was discovered using dRNA-Seq, where in the absence of any known bias resulting in a false TES, a single mapped read was deemed confirmatory. To avoid non-specific priming, reads with three or more mapped A letters on their 3′ end were discarded from the cDNA dataset. Reads with poly(A) tails on both of their ends were discarded, except for complex transcripts, for which the previously annotated TES was considered. Reads with a larger than 10 nt difference in their 5′ or 3′ ends were considered novel length isoforms (L: longer 5′ UTR, S: shorter 5′ UTR, AT: alternative 3′ termination). Short length isoforms harbouring a truncated version of the known open reading frame (ORF) were considered novel putative coding transcripts, and designated as ‘0.5’. Short transcripts without an ORF and with a TES upstream of the coding transcript’s 3′ end were designated novel 3′-truncated (TR) non-coding transcripts. Long reads spanning at least two known transcripts with different directions were named complex transcripts (C). We assume that these complex transcripts start at the closest upstream annotated TSSs. Splice junctions were accepted if the intron boundary consensus sequences (GT and AG) were present in at least two sequencing reads, or were confirmed either by dRNA-Seq or by PCR analysis. Promoters and initiation sites were discovered using MEME^29^. Possible protein products were predicted by aligning ORFs of putative non-coding RNAs to online databases using the BLASTP suit^30^ with an expected threshold of 10. Reads were visualized using the Geneious software suite^31^ and IGV.

### PCR analysis

PCR analysis and polyacrylamide gel electrophoresis was performed for validating antisense transcripts and splicing events. SuperScript III Reverse Transcriptase (Life Technologies) enzyme, 70 ng of total RNA and gene specific primers were used for the cDNA synthesis, according to the manufacturer’s instructions. A noRT control was used for testing the potential DNA contamination. The cDNA samples were amplified using the Applied Biosystem’s Veriti Thermal Cycler with KAPA HiFi PCR Kit (KAPA Biosystems) according to the manufacturer’s recommendations. The running conditions were as follows: 3 min at 95 °C for initial denaturation, followed by 35 cycles at 98 °C for 20 s (denaturation), at 63 °C for 20 s (annealing), and at 72 °C for 2 min (extension). Final elongation was set at 72 °C for 5 min. Primers used in this study are outlined in Supplementary Table S1. For amplicon separation and visualization, a 12% polyacrylamide gel was prepared. Lanes were loaded with either GeneRuler Ultra Low Range DNA Ladder (Thermo Fisher Scientific), or the samples. Staining was performed with GelRed (Biotium).

### Short-read sequencing data acquisition and analysis

In order to compare short-read sequencing data of the AcMNPV transcriptome to our own long-read sequencing data, the single end Illumina sequencing reads deposited in the Sequence Read Archive under accession SRA057390 were retrieved. The reads were aligned to the reference genome of AcMNPV strain E2 (GeneBank accession: KM667940.1) using TopHat v. 2.1.1^32^. Reads were visualized using IGV.

## Results

### Analysis of the AcMNPV transcriptome with long-read sequencing

**Figure 1:**
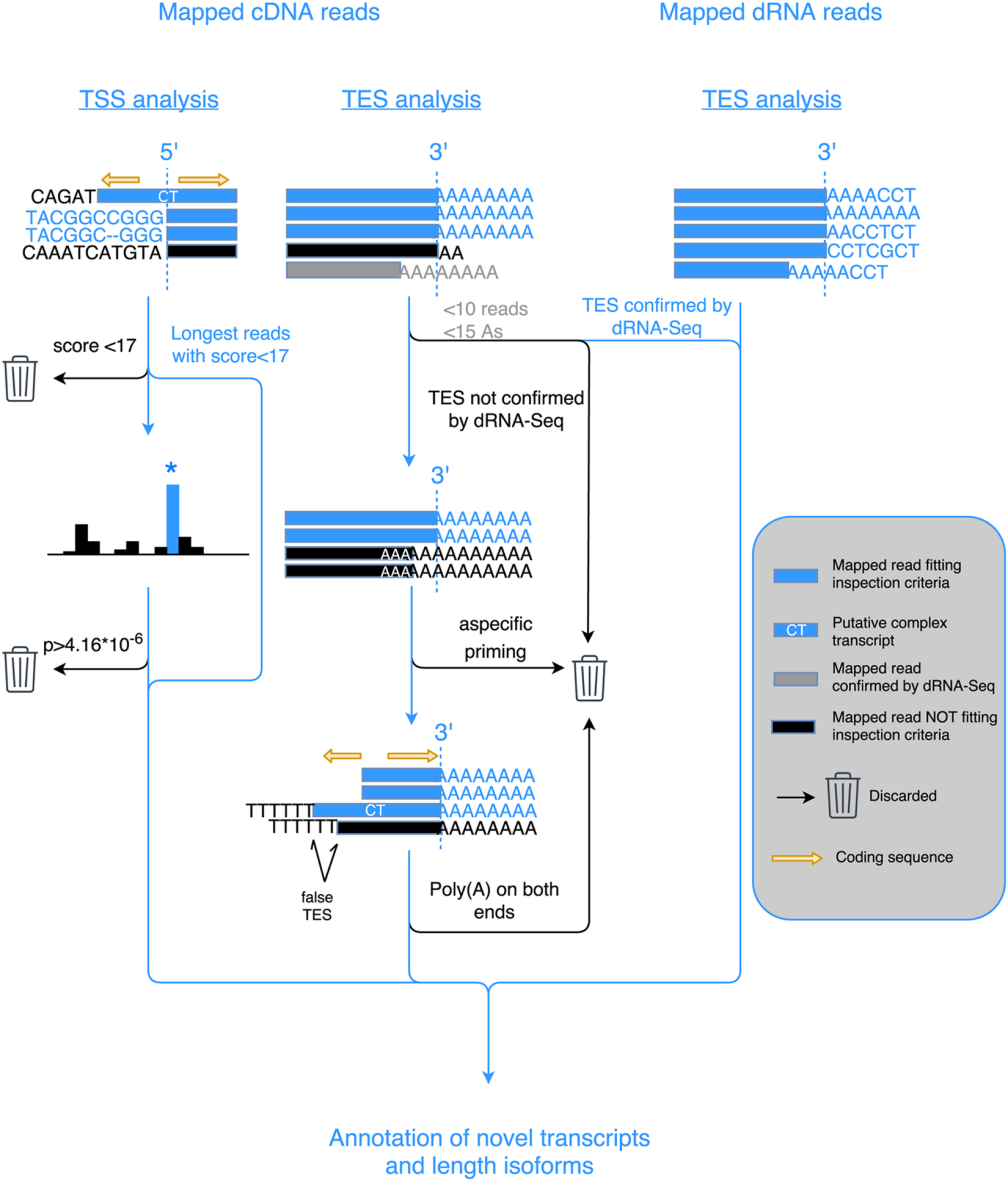
The schematic representation of the workflow used to annotate novel transcripts. Only the cDNA-sequencing reads were used for identifying TSSs. The read start distribution was obtained by only counting reads which contained the 5′ adaptor sequence. Nucleotide positions where the read start distribution was significantly different from a Poisson distribution were annotated as TSSs. In the case of complex transcripts, if no TSS could be determined, the closest upstream TSS was assumed to be the TSS. Both dRNA and cDNA reads were used to identify TESs. In case of the dRNA-Seq, support by one polyA-containing read was sufficient to annotate a TES, while in the case of cDNA-Seq, at least 10 reads were required to support a given TES, and also reads that could have arisen through non-specific priming, and reads containing poly(A) sequences on both ends were discarded from the TES analysis. The complex transcripts, supported by reads with double polyA-tails, were annotated, and their orientation was determined according to the polyA-tail, which passed the TES annotation criteria.

**Figure 2:**
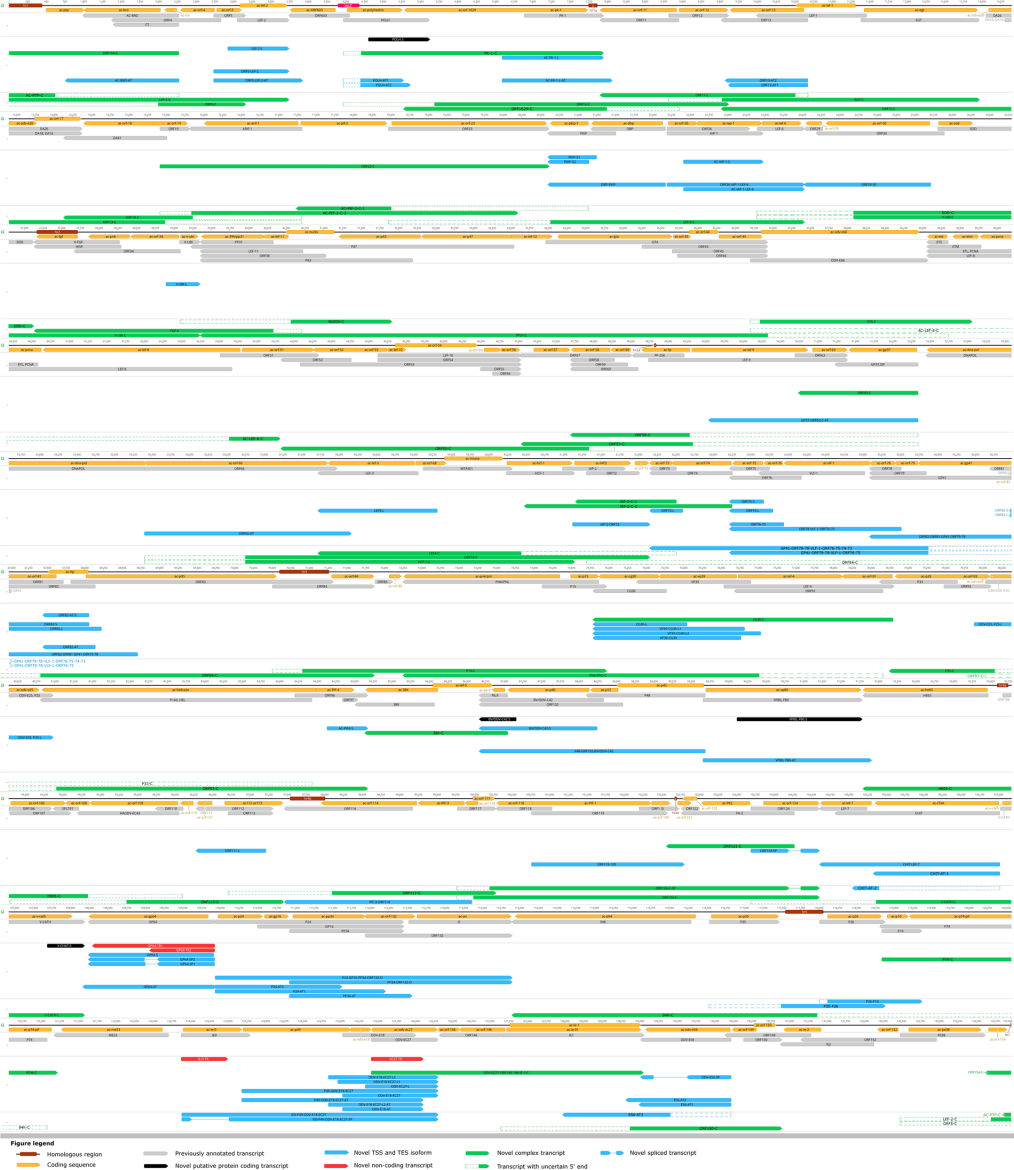
Location of the previously and newly annotated transcripts on the linear view of the AcMNPV genome. Colour code: brown rectangles: homologous regions; yellow arrow-rectangles: coding sequences; grey arrow-rectangles: previously annotated transcripts; black arrow-rectangles: novel putative protein coding transcripts; blue arrow-rectangles: novel TSS and TES isoforms and novel polycistronic transcripts; red arrow-rectangles: novel non-coding transcripts; green arrow-rectangles: novel complex transcripts, purple rectangle: lacZ gene inserted in to the genome. Transcripts with undetermined TSSs were hypothesized to start at the closest upstream TSS in the same orientation, and their missing segment is marked by dashed contours.

**Figure 3:**
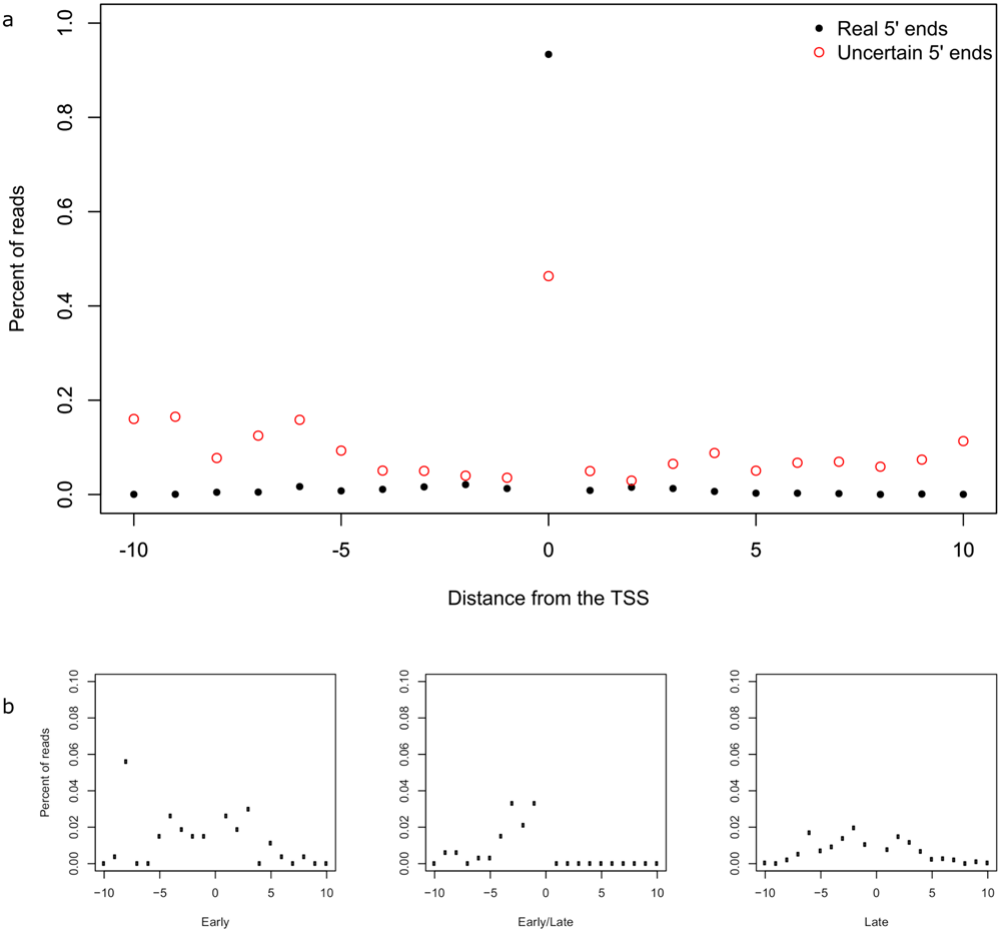
TSS variations. Panel a shows the frequency of reads starting in the vicinity of TSSs for real 5′ ends (black dots) and in case of uncertain 5′ ends (red circles). Panel b shows the frequency of reads starting in the vicinity of real TSSs of temporally different expression. The salient values of the TSS positions are not shown.

In this study, we carried out ONT MinION cDNA and direct RNA sequencing analysis (Fig. 1) of the AcMNPV transcriptome (Fig. 2). The cDNA-Seq yielded 324,677 reads of which 103,133 mapped to the AcMNPV genome (the rest was mapped to the transcripts of the host cell), with an average read length of 1,053 bp and an average genome coverage of 510. The dRNA-Seq technique yielded 6,482 reads, 2,430 mapping to the viral genome, with an average read length of 614 bp and an average genome coverage of 10. In this work, we detected and annotated altogether 132 novel RNA molecules, including 80 full-length transcripts and 46 transcripts with undetermined TSSs (Supplementary Table S2). We identified five novel splice isoforms (Supplementary Table S3). Additionally, we determined the TSSs of 64 and the TESs of 113 earlier reported transcripts with base-pair precision. The MinION cDNA sequencing technique allows the precise identification of TSSs, although similarly to other techniques, it is also afflicted by sample degradation. Another issue arises when more than three nucleotides of the MinION strand switching oligonucleotide is found on the viral genome, allowing both template switching and false priming. These events resulted in a total of 154 probably false 5′ ends, of which 47 belong to novel transcripts or transcript isoforms, including 38 complex transcripts, 6 polycistronic transcripts, and 3 transcripts with alternative termination. The 5′ ends of these transcripts were annotated as undetermined TSSs. False 5′ ends, marked by the absence of the MinION strand switching oligonucleotide, show a much higher variation around the TSS than real TSSs (Fig. 3, panel a), which is probably caused by the frequent template switching events, and demonstrates the utility of our workflow for distinguishing between false and real TSSs. It has been previously shown^33^ that the TSSs of the transcripts vary with a few nucleotides. In our annotation, we chose a position as TSS, where significantly more reads started than expected in its surrounding 101 bp region. Out of the 655 positions where reads with real 5′ ends started, 101 were accepted as TSSs (p < 4.16 * 10^−6^, Bonferroni 0.05/101/119), of which 79 belonged to novel transcripts. The remaining positions were considered to be the result of TSS variation and RNA degradation. Template switching and false priming errors contribute to the artefactual 3′ ends of the reads, where the oligo(dT) primers, hybridize with homologous stretches of the transcripts, generally with a much lower affinity. Out of the 23,261 3′ end positions, we found 496 where the upstream genomic region contained at least three A letters, which were removed from further analysis. We discovered 45 novel positions with no evidence of template switching or false priming, thus these were considered as novel TESs.

### Analysis of the viral transcriptome using short-read sequencing

In order to compare short- and long-read sequencing of the viral transcriptome, we retrieved and aligned the Illumina sequencing data produced by Chen and coworkers^3^. In total 188,025,259 reads mapped to the AcMNPV genome.

### Novel putative protein-coding genes

The putative protein-coding genes identified in this work are all 5′-truncated forms of previously annotated genes. These transcripts share common PA signals with their host genes. In total, we discovered four novel putative mRNAs, three of these starting from the second nucleotide of the canonical LIS, TAAG (the underlined A letter is the first nucleotide of the transcripts). BV/ODV-C42.5, V-CHAT.5, and P80.5 feature the same initiator as their non-truncated forms implying their late transcription. This is indicated by the absence of canonical TATA promoters in these transcripts. The TSS of POLH.5 is located in a ACAGG motif, which is similar to the previously described arthropod initiator element ACAGT^34,35^ except that it is not preceded by a canonical TATA motif. The presence of this initiator indicates that an 819 bp long 5′ truncated POLH may be transcribed by the host RNAP. Regarding their TESs, all novel putative protein-coding transcripts contain one or more PASs upstream their 3′ ends, starting at an average distance of 16.2 bp.

### Novel non-coding transcripts

In this study, we identified four novel non-coding transcripts, all being 3′ truncated forms of already known mRNAs. BLASTP analyses of the transcripts’ ORFs show no homology with known proteins. These transcripts start from the same promoter (all from the LIS) as their host genes, as in GP64-TR1, GP64-TR2, IE-0-TR and EC27-TR transcripts. These RNA molecules have the same TSS as their full length transcripts, but they lack stop codons and thus ORFs. The gene *gp64* is expressed at both early and late stages of the viral infection, which is indicated by the presence of two TATA promoters upstream it’s TSS. Unlike GP64-TR2, GP64-TR1 has no canonical PAS, instead the transcript ends at a homopolymer T comprised of four nucleotides. This can act as a polyadenylation signal for the viral RNA polymerase^7^.

The IE-0 is the non-spliced version of the IE-1 transcript, and encompasses the *ie0* gene of the virus^36^, but is expressed during the late phase. IE-0-TR has the same TSS as IE-0, and harbours a PAS 17 bp upstream of its TES. The promoter consensus sequences of the novel 3′ truncated transcripts are identical to those of their full length variants, albeit the presence of four T letters adjacent to the TES of GP64-TR2 suggests a late transcription.

### TSS and TES isoforms

Transcriptional start site isoforms differ in the length of their 5′UTRs from each other. Using long-read sequencing, we could identify 23 novel TSS variants. Twenty-one of these start at the LIS (Supplementary Fig. S1), like their previously annotated isoforms, except two transcripts, ORF73 and EC27, which initiate at a consensus TAAG, but their longer isoform OFR73-L and EC27-L is missing this sequence or a canonical TATA motif. ORF111 was previously characterized as an early gene^3^, but its longer isoform, ORF111-L starts at the LIS, and contains two upstream TATA boxes at 24-bp and 41-bp distance. Additionally, LEF-3 and its longer isoform LEF-3-L both initiate at a slightly modified version of the arthropod initiator: CATT and CAAT respectively. We found that five out of the late TSS isoforms contain canonical TATA motifs at an average of 12.4 bp upstream their TSS, three of which (ORF75-L, ORF82-L and VP39-CG30-L1) are adjacent to the LIS. The shorter isoform of GP64, designated GP64-S, contains both a late initiator and a TATA box, which is consistent with the early and the late transcription of its full length isoform. A comparison of the TSS variations of the three kinetic classes shows that early and early/late mRNAs tend to vary more around their most abundant start sites than late transcripts, (SD_E_ = 0.014, SD_E/L_ = 0.01, SD_L_ = 0.006, Fig. 3, panel b), which is to be expected due to the LIS-dependent initiation of late transcripts. Transcripts with uncertain 5′ end were labelled as starting at the closest upstream TSS, because we assume that they are controlled by the corresponding upstream promoters.

**Figure 4:**
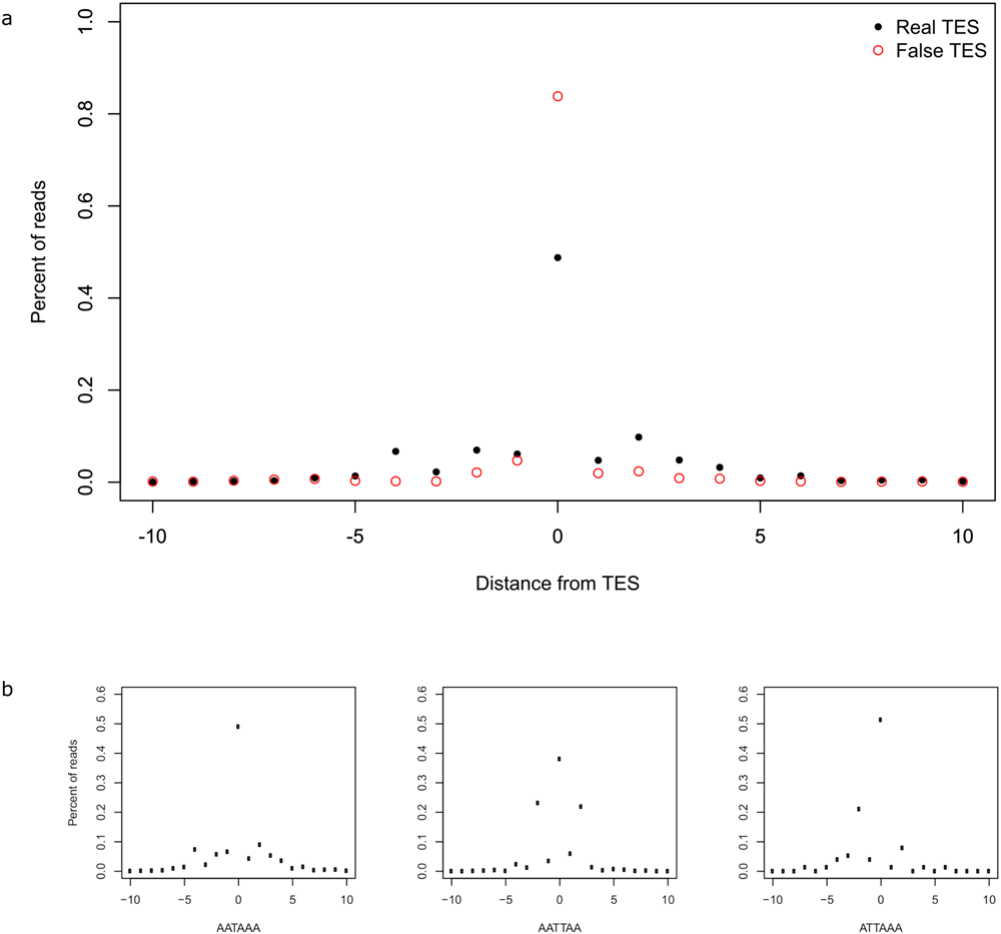
TES variations. Panel a shows the frequency of reads ending in the vicinity of TESs for real 3′ ends (black dots) and in case of uncertain 3′ ends (red circles). Panel b shows the frequency of reads ending in the vicinity of real TESs for the first three most common PASs.

Transcriptional end site isoforms differ in the 3′ UTRs compared to the formerly annotated transcripts. In this study, we detected twenty-four TES variants. Twelve of these have canonical PASs, while six of the transcripts are terminated at a T-rich region with an average T count of 2.5 (Supplementary Table S4). This and the presence of a LIS at their TSS suggest that they are probably transcribed by the viral RNAP. The same may be true for AC-PK-1-L-AT, the single TES isoform out of the nine without a canonical PAS, but starting at a LIS and terminating at a region composed of 3 T letters. We found that the real 3′ ends show a greater variation around the TES compared to false read endings (Fig. 4 panel a). We also demonstrated that the variance in the TES locations depends on the sequence composition of the upstream PAS (Fig. 4 panel b).

### Novel splice isoforms

We identified five novel splice isoforms and confirmed the existence of three previously described spliced transcripts, all with a consensus GT at the splice donor site and AG at the splice acceptor site (Supplementary Table S3). A novel splice variant of ODV-E56, designated ODV-E56-SP, was confirmed by dRNA-Seq, while the other novel splice isoforms were confirmed using PCR analysis followed by PAGE (Supplementary Fig. S2). We detected all of the novel splice sites in the Illumina short-read sequencing datasets, but they represented less than 0.1% of the reads in the given region, which is below the detection threshold of 5 RPKM used by Chen *et al*.^3^. The low abundance of these spliced transcripts is also indicated by the weak bands corresponding for the spliced amplicons in lane A and B of the PAGE. Splicing causes frame-shift in ODV-E56 and GP64-SP2 resulting in putative 3′ truncated, 191 and 261 amino acid long peptides. Further investigations are needed in order to reveal the functions of these transcripts, if they have any.

### Polycistronic transcripts

This study revealed extensive polycistronism in the AcMNPV transcriptome. Altogether, twelve bicistronic, four tricistronic, three tetracistronic, three pentacistronic, and a single septacistronic transcripts have been detected by long-read sequencing. Ten of these transcripts share their TSSs with the first genes of the polycistronic unit that are also expressed as monocistronic RNA molecules. Five of the polycistronic transcripts contain novel TSSs. These transcripts contain LISs and are terminated at an average distance of 23.3 bp downstream of a canonical PAS. The ORF29-30 starts at a CAGT motif and contains a PAS 21 bp upstream its TES. Nineteen of the detected polycistronic transcripts have the same TES as the most downstream gene expressed as monocistronic transcripts.

### Complex transcripts

The complex transcripts are special polycistronic RNAs with genes standing in opposite orientations. In this work, we identified forty-nine complex transcripts, ten of which start at a LIS, and three of them have a canonical TATA promoter at an average distance of just 2.6 bp upstream. One isoform of ORF124-C contains the same splice site as ORF124-SP, and thus has been tagged ORF124-C-SP. Four of the complex transcripts overlap HR1, a homologous region of the AcMNPV genome, which serves as replication initiation site^37^. We detected four complex transcripts that overlap the hr1, a putative origin of replication of AcMNPV.

### Novel transcriptional overlaps

**Figure 5:**
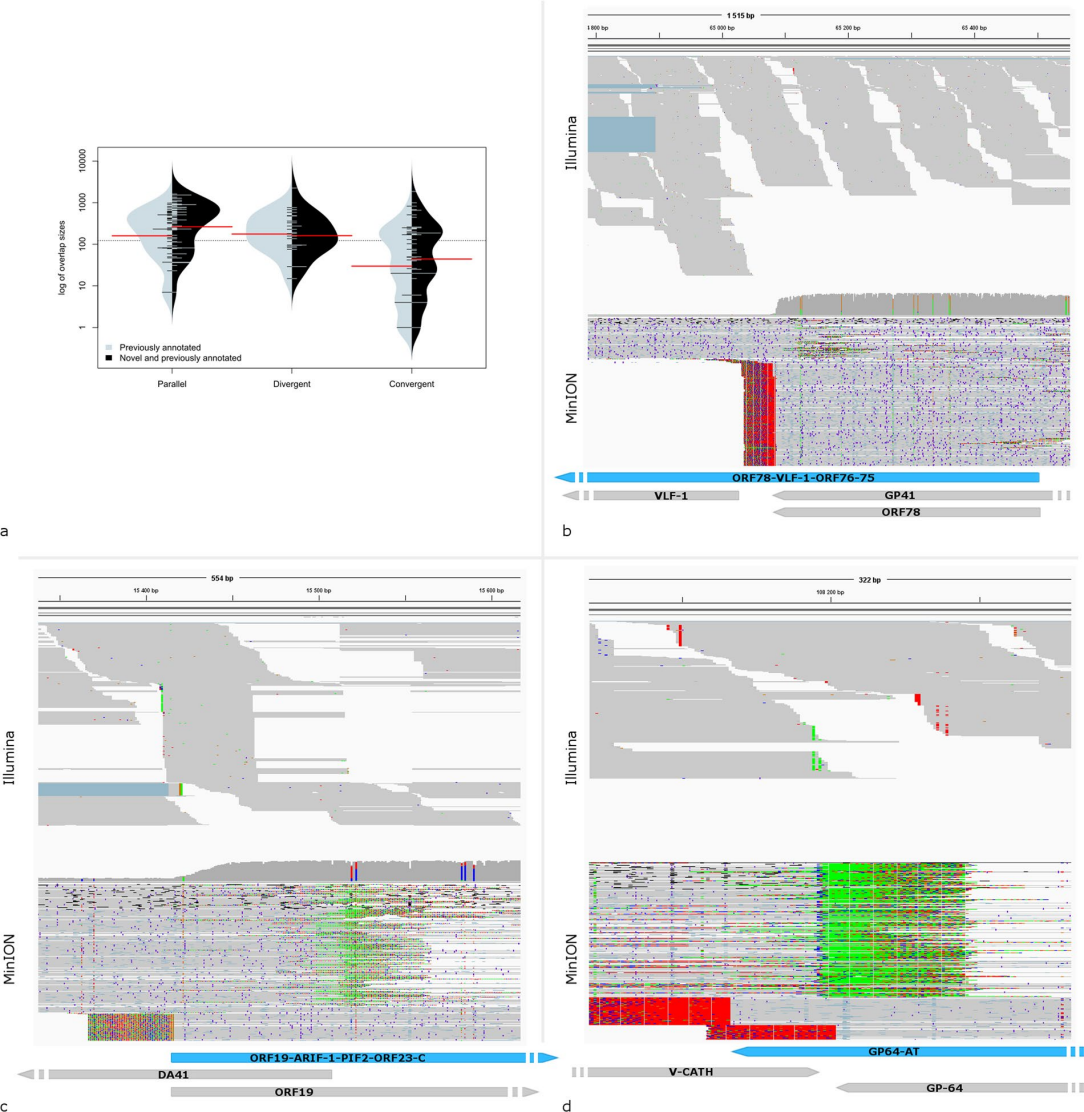
Size distributions and examples for the transcriptional overlaps. Panel a shows the comparison of size distributions of transcriptional overlaps of previously annotated transcripts (grey) versus previously annotated and novel transcripts with a certain TSS (black). Colour code: The red lines represent the mean values. Panel b Parallel; Panel c Divergent, and Panel d Convergent overlaps of previously annotated and novel transcripts. Reads from the Illumina and MinION cDNA sequencing were visualized using IGV, and their schematic representation. Grey arrow-rectangles represent previously annotated transcripts, while blue arrow-rectangles represent novel transcripts. The annotations were cut to fit the representation, indicated by a non-continuous annotation on one side.

The viral transcripts can overlap in parallel (tail-to-head), convergent (tail-to-tail) or divergent (head-to-head) manners. Our study revealed a complex meshwork of transcriptional read-throughs in AcMNPV. We discovered 72 parallel, 37 convergent and 8 divergent overlaps of the previously annotated and novel transcripts, doubling the number (105 previously annotated and 117 novel) and increasing the average size (228.59 bp and 414.7 bp respectively) of transcriptional overlaps (Supplementary Table S5 and Fig. 5). We identified 98 additional overlaps (34 parallel, 31 divergent, 33 convergent) where one of the TSSs of the partner transcripts was only predicted. Comparison of the short-read sequencing data to our long-read sequencing data revealed that the overlapping reads between adjacent transcripts can be found in both datasets, however especially in the case of the parallel overlaps (Fig. 5b), the identification of the TSSs in the overlapping region using short reads is nearly impossible.

## Discussion

In this study, we used the Oxford Nanopore Technologies’ MinION long-read sequencing platform for the characterization of the transcriptional landscape of a baculovirus. Our investigations revealed a much higher complexity of the viral transcriptome than it had been formerly described. Using both cDNA and direct RNA sequencing methods, we have identified 132 novel transcripts and transcript isoforms, most of which overlap already known transcripts. The longer TSS and TES variants either form a new overlap or they increase the extent of the overlaps formed by the shorter transcript isoforms. No complex transcripts have been described in baculoviruses before this study. These RNA molecules together with the polycistronic transcripts represent very long transcriptional overlaps. Polycistronism is typical in the bacterial genes, however, this phenomenon is extremely rare in eukaryotic organisms. Some eukaryotic viruses solved the problem of reading multiple messages from a single RNA molecule using various mechanisms, such as leaky ribosomal scanning (in retroviruses^38^ and papillomaviruses^39^), ribosomal frameshifting (retroviruses^40^) or utilizing internal ribosome entry sites (in picornaviruses^41^). However, no such mechanisms have been described in baculoviruses so far, therefore only the most upstream genes of the RNA molecules will be translated. To address the question of multiple ORFs translating from polycistronic mRNAs there is a need for further studies, such as ribosome profiling^42^. The question can thus be raised about the role of the multigenic RNA molecules in the viral pathogenesis, if there is any. Theoretically, it is possible that these long transcripts represent transcriptional noise. It is also possible these RNA molecules are precursors for smaller regulatory molecules or that they have post-transcriptional function. We can speculate whether these molecules are mere by-products of a transcriptional read-through mechanism whose function is to regulate gene expression through the collision and/or competition between the transcription machineries of adjacent and distal genes. Indeed, the phenomenon of transcriptional interference between the convergent gene pairs have been described in other organsisms^43,44^. These interactions may form a genome-wide meshwork for the regulation of gene expression designated as transcriptional interference network^45^. We also identified four complex transcripts overlapping one of the replication origins (hr1) of the baculovirus, which suggest a potential interaction between the transcription and replication machineries. In this study, we also demonstrated that the TSS isoforms have the same types of promoters and initiator regions, suggesting that the expression kinetics of the longer and the shorter transcripts of a given gene are the same. In contrast, a different promoter or initiator motif may mean an uncoupled transcription of the mRNA isoforms, which could be the result of random processes, but can serve regulatory purposes, such as differential gene expression. We annotated promoters and ORFs for the novel 5′-truncated mRNAs, which therefore may encode protein molecules, but we cannot exclude that they are long non-coding RNAs, or are precursors for short non-coding RNAs. Additionally, we demonstrated the significance of template switching and false priming on transcript isoform annotation using the ONT long-read sequencing technology. While it was formerly considered that these effects can play a role in false TES annotation in the transcripts containing homopolymer-A stretches^22^ upstream their 3′ ends, our results suggest that the presence of homology between the 5′-end adapter and the transcripts can also result in a number of false transcription reads.

### Data availability

The sequencing data and the transcriptome assembly have been uploaded to the European Nucleotide Archive under the project accession number PRJEB24943.

## Electronic supplementary material

Supplementary Figures

Supplementary Tables
