# Supplementary material for: Third-generation Sequencing Reveals Extensive Polycistronism and Transcriptional Overlapping in a Baculovirus
Source: Sci Rep. 2018 Jun 5;8:8604. doi: 10.1038/s41598-018-26955-8 (PMC5988703; doi:10.1038/s41598-018-26955-8)
Supplement: Supplementary file 1 — Supplementary Figures [file 41598_2018_26955_MOESM1_ESM.pdf]

Third-generation Sequencing Reveals Extensive Polycistronism and Transcriptional Overlapping in a Baculovirus

Supplementary material

Norbert Moldován[1], Dóra Tombácz[1], Attila Szűcs[1] , Zsolt Csabai[1] , Zsolt Balázs[1] , Emese Kis[2] , Judit Molnár[2] , Zsolt Boldogkői[1]*

1 Department of Medical Biology, Faculty of Medicine, University of Szeged, Szeged, 6720, Hungary
2 Solvo Biotechnology, Szeged, 6720, Hungary

* To whom correspondence should be addressed

Tel: +36-62- 545-595
Fax: +36-62- 545-131

E-mail: boldogkoi.zsolt@med.u-szeged.hu

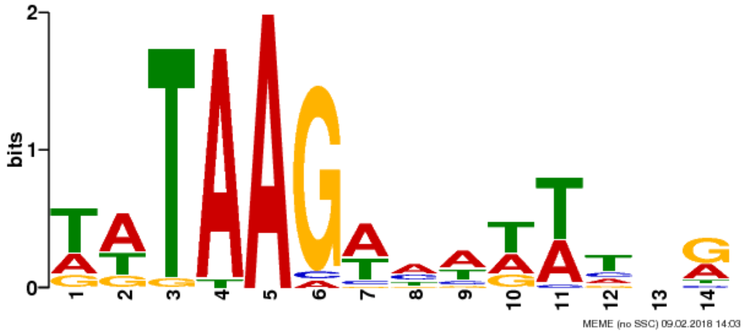

Supplementary figure S1 The late initiator motif of novel transcriptional start site isoforms. The motif was discovered using the MEME suit [1] with an e-value of $2.7*10^{-5}$, with a log likelihood ratio of 145.

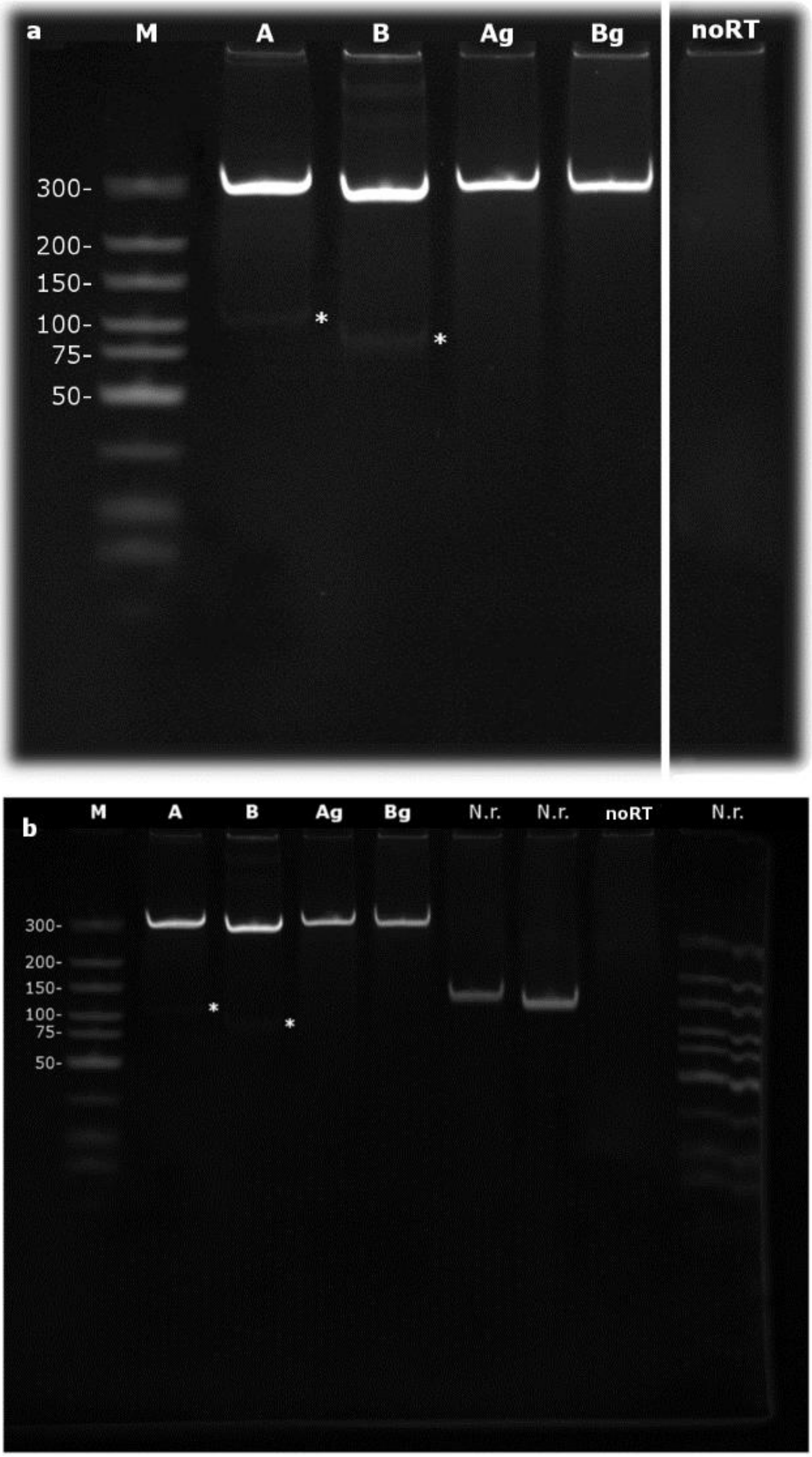

a
M
A
B
Ag
Bg
noRT
300-
200-
150-
100-
75-
50-
*
*
b
M
A
B
Ag
Bg
N.r.
N.r.
noRT
N.r.
300-
200-
150-
100-
75-
50-
*
*

Supplementary figure S2 a. 12% polyacrylamide gel electrophoresis of splice isoforms and antisense transcripts. Lanes A and B were loaded with cDNA products from PCR of ORF124-SP and GP64SP1 and –SP2 respectively, lanes Ag and Bg were loaded with products of amplified gDNA using the same primers as in case of lanes A and B. Lane M was loaded with the molecular weight size marker GeneRuler Ultra Low Range DNA Ladder (Thermo Fisher Scientific), while the lane noRT was loaded with noRT samples to detect DNA contamination. On lanes A and B two weak bands can be detected (marked with an *) resembling the spliced versions of ORF124 (product size:108 bp) and GP64 (product size:89 bp in case of SP1 and 81 bp in case of SP2). Two bands with high contrast appear at the same level on lanes A and Ag and B and Bg resembling the non-spliced versions of the transcripts. The image was overexposed to make marked bands more visible. Two lanes not related to our results were cropped from the image and lane noRT was placed adjacent to lane Bg. **b.** The same gel photo in its original form and labelled. Lanes N.r. are not related to the current results.

Supplementary datasets

Supplementary Table S1 Primers used for detecting splice isoforms, and amplicon sizes produced during PCR. **Supplementary Table S2** The novel transcripts and transcript isoforms detected and annotated in this study. Transcripts detected in both cDNA and dRNA sequencing are marked with a ✓. The p-values of 5’ ends for transcripts with a real TSS are to be found in the column titled “5’ end p values”. For transcripts with an uncertain TSS the most abundant 5’ end detected in the sequencing data is presented.

Supplementary Table S3 Novel and previously annotated spliced transcripts.

Supplementary Table S4 The promoters, initiators, T-rich termination and polyadenylation signals of novel transcripts and transcript isoforms. The start and end positions of each motif is separated by a semicolon (;) while in case of the presence of multiple motifs they are separated by a slash (/).

Supplementary Table S5 Parallel, divergent and convergent overlaps of the novel and previously annotated transcripts with the size of the overlaps. The overlaps between the transcripts where one of the partners contains undetermined TSS are marked with ‘*’.

Reference Used in the Supplementary Material

1. Bailey, T. L. & Elkan, C. Fitting a mixture model by expectation maximization to discover motifs in biopolymers. Proceedings. Int. Conf. Intell. Syst. Mol. Biol. 2, 28–36 (1994).
